# Rural–Urban differences in Use of Rhythm Control Therapies in Patients with Incident Atrial Fibrillation: A Finnish Nationwide Cohort Study

**DOI:** 10.3390/ijerph191811191

**Published:** 2022-09-06

**Authors:** Konsta Teppo, Jussi Jaakkola, Fausto Biancari, Olli Halminen, Miika Linna, Jari Haukka, Jukka Putaala, Pirjo Mustonen, Janne Kinnunen, Alex Luojus, Saga Itäinen-Strömberg, Juha Hartikainen, Aapo L. Aro, K. E. Juhani Airaksinen, Mika Lehto

**Affiliations:** 1 Faculty of Medicine, University of Turku, 20500 Turku, Finland; 2 Heart Unit, Satakunta Central Hospital, 28500 Pori, Finland; 3 Heart and Lung Center, Helsinki University Hospital, University of Helsinki, 00014 Helsinki, Finland; 4 Department of Industrial Engineering and Management, Aalto University, 02150 Espoo, Finland; 5 University of Eastern Finland, 70211 Kuopio, Finland; 6 University of Helsinki, 00014 Helsinki, Finland; 7 Neurology, Helsinki University Hospital, University of Helsinki, 00014 Helsinki, Finland; 8 Heart Center, Turku University Hospital, 20014 Turku, Finland; 9 Heart Center, Kuopio University Hospital, 70210 Kuopio, Finland; 10 Department of Internal Medicine, Lohja Hospital, Lohja, Finland

**Keywords:** atrial fibrillation, rural–urban disparities, rhythm control therapies, antiarrhythmic drugs, cardioversion, ablation

## Abstract

**Background:** Rural–urban disparities have been reported in the access, utilization, and quality of healthcare. We aimed to assess whether use of antiarrhythmic therapies (AATs) in patients with atrial fibrillation (AF) differs between those with rural and urban residence. **Methods:** The registry-based FinACAF cohort covers all patients with AF from all levels of care in Finland. Patients were divided into rural and urban categories and into urbanization degree tertiles based on their municipality of residence at the time of AF diagnosis. The primary outcome was the use of any AAT, including cardioversion, catheter ablation, and fulfilled antiarrhythmic drug (AAD) prescription. **Results:** We identified 177,529 patients (49.9% female, mean age 73.0 (SD13.0) years) with incident AF during 2010–2018. Except for AADs, the differences in AAT use were nonsignificant when patients were stratified according to the rural–urban classification system (urban vs. rural adjusted incidence rate ratios (aIRRs) with 95% CIs for any AAT 1.01 (0.99–1.03), AADs 1.11 (1.07–1.15), cardioversion 1.01 (0.98–1.03), catheter ablation 1.05 (0.98–1.12)). However, slightly higher use of all rhythm control modalities was observed in the highest urbanization degree tertile when compared to the lowest tertile (aIRRs with 95% Cis for any AAT 1.06 (1.03–1.08), AADs 1.18 (1.14–1.23), cardioversion 1.05 (1.02–1.08), catheter ablation 1.10 (1.02–1.19)). **Conclusions:** This nationwide retrospective cohort study observed that urban residence is associated with higher use of AADs in patients with incident AF. Otherwise, the observed disparities were only marginal, suggesting that in the use of rhythm control therapies, no large rural–urban inequity exists in Finland.

## 1. Introduction

Atrial fibrillation (AF), the most common sustained arrhythmia with a prevalence as high as 4.1%, is associated with increased risk of ischemic stroke and mortality [1,2]. Symptoms related to AF range from none to disabling, often impacting daily life with exercise intolerance, fatigue, and palpitations [3]. Although rate control approach is often sufficient to improve AF-related symptoms, certain aspects clearly support electing rhythm control strategy, referring to attempts to restore and maintain sinus rhythm. Rhythm control strategy may encompass a combination of antiarrhythmic therapies (AATs), including catheter ablation, cardioversion, and antiarrhythmic drugs (AADs) [3]. While arrhythmic symptoms are the primary indication for AATs in current guidelines, recent findings have also suggested outcome benefits of rhythm control strategy [4,5,6].

Previous studies have revealed that individuals residing in rural areas have worse outcomes in cardiovascular diseases and higher all-cause mortality [7,8,9]. Likewise, rural–urban inequalities have been observed in the access, utilization, and quality of healthcare [10,11,12,13]. However, in patients with AF, prior research on treatment and outcome disparities between rural and urban areas is limited and has shown somewhat inconsistent results [14,15,16,17,18]. This nationwide cohort study covering all patients with incident AF in Finland aimed to assess whether the use of rhythm control therapies varies between patients residing in rural and urban areas.

## 2. Methods

### 2.1. Study Population

The Finnish AntiCoagulation in Atrial Fibrillation (FinACAF) Study (ClinicalTrials Identifier: NCT04645537; ENCePP Identifier: EUPAS29845) is a nationwide historic cohort study covering all patients with AF in Finland during 2004–2018 [2]. The study sample was gathered from interlinked national health care registers (hospitalizations and outpatient specialist visits: HILMO; primary health care: AvoHILMO; and National Reimbursement Register upheld by Social Insurance Institute: KELA). All individuals with an International Classification of Diseases, Tenth Revision (ICD-10) diagnosis code I48 (including atrial fibrillation and atrial flutter, together referred as AF) recorded between 2004–2018 were included in the cohort and cohort entry was considered to occur on the date of the first recorded AF diagnosis. The exclusion criteria were age < 20 years at AF diagnosis and permanent migration abroad before 31 December 2018. Follow-up continued until 31 December 2018 or death, whichever occurred first. The current sub-study was conducted within a cohort of patients with incident AF, established in previous studies of the FinACAF cohort [19,20,21]. In this cohort, patients with a recorded AF diagnosis during 2004–2006 were excluded because the 2-year medical history was considered too short to exclude the presence of an AF diagnosis before the cohort entry. Additionally, patients who had fulfilled an OAC prescription during 2004–2006 or within a year before the date of first AF diagnosis were excluded, since most of them likely had a previous diagnosis of AF. Furthermore, patients entering the cohort before the introduction of AF specific ablation codes in 2010 were excluded. The patient selection process is summarized in Supplementary Figure S1.

### 2.2. Rural–Urban Status

The patients were categorized to rural and urban groups according to Finland’s Environmental Administration’s rural–urban classification system and patients’ municipality of residence at cohort entry. In this classification, several variables, such as population, labor, building, and road network data, are used to define areas rural–urban status, and urban municipalities have a center with more than 15,000 residents [22]. Additionally, patients were divided into tertiles according to the degree of urbanization of their municipality of residence, acquired from Statistics Finland [23]. The degree of urbanization refers to the proportion of people in a municipality living in localities or urban settlements.

### 2.3. Use of AATs

As an indicator of a pursuit of rhythm control strategy, the first-ever use of any AAT was the primary outcome of the study, including recorded cardioversion (Nordic Classification of Surgical Procedure (NCSP) codes: TPF20, WVA50, WX904), catheter ablation (NCSP codes: TPF44, TPF45, TPF46), and claimed AAD prescription (ATC code C01B antiarrhythmics class I and III, plus ATC code C07AA07 sotalol). The outcome was considered to occur on the date of first AAD purchase or procedure date, whichever occurred first. The secondary outcomes were redeemed AAD prescription, as well as cardioversion and catheter ablation procedures individually.

### 2.4. Statistical Analysis

The chi-square test was used to compare differences between proportions, and the independent samples *t*-test and analysis of variance to analyze continuous variables. Poisson regression was used to estimate the adjusted and unadjusted incidence rate ratios of AATs. The Poisson regression models were adjusted for age (categorical variable in 10-year groups), gender, calendar year of AF diagnosis, income quartiles, educational level, dementia, cancer, alcohol use disorder, psychiatric disorders, prior stroke, abnormal liver function, abnormal kidney function, diabetes, hypertension, coronary artery disease, and heart failure. The definitions of the comorbidities are displayed in Supplementary Table S1. Statistical analyses were performed with the IBM SPSS Statistics software (version 27.0, SPSS, Inc., Armonk, NY, USA) and R (version 4.0.5, https://www.R-project.org (accessed on 1 March 2022)).

## 3. Results

We identified 177,529 patients (49.9% female, mean age 73.0 (SD13.0) years) with incident AF during 2010–2018, the mean follow-up being 2.6 (SD 2.5) years. Patients with rural residence had lower educational and income levels and higher prevalence of cardiovascular comorbidities than patients with urban residence (Table 1).

### 3.1. Use of Any Rhythm Control Therapy

**Figure 1:**
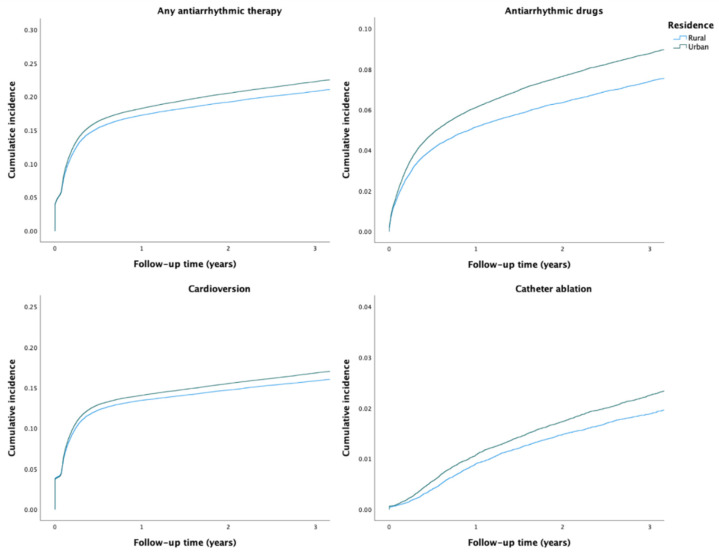
Crude cumulative incidence curves of the use of AATs.

**Figure 2:**
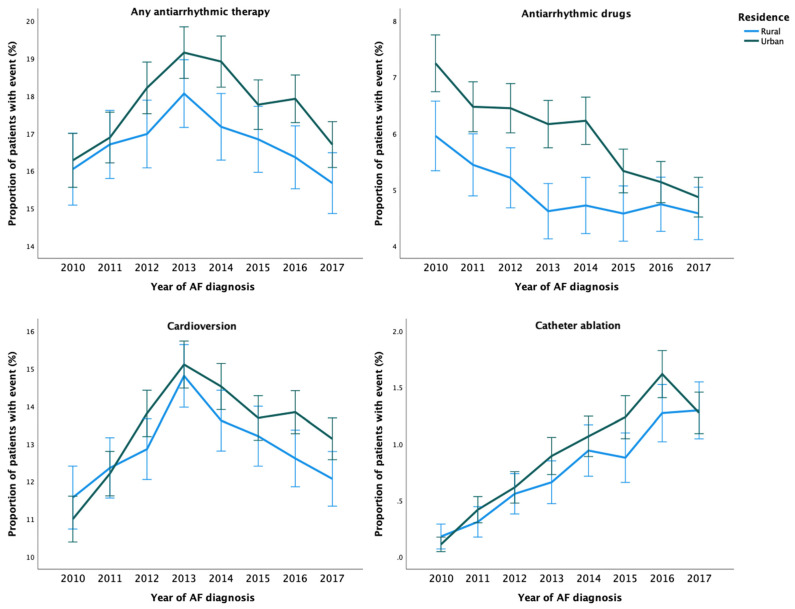
Temporal trends in the proportion of patients with AATs within one-year follow-up from cohort entry according to the year of AF diagnosis.

During the study period, any AAT was used in 36,668 (20.7%) patients. The crude incidence of any AAT was higher among patients with urban residence than among those with rural residence (Table 2 and Figure 1). After adjustments, no disparity in the rate of any AAT use was observed based on the rural–urban classification system. However, the highest urbanization degree tertile was associated with higher rate of AAT use, when compared to the lowest tertile (Table 2). Use of any AAT during the first year after AF diagnosis was consistently lower across the study period in patients with rural residence (Figure 2).

### 3.2. Antiarrhythmic Drugs

A total of 14,043 (7.9%) patients received AADs during the study period. The unadjusted and adjusted incidence of AAD use were higher in patients with urban residence and higher urbanization degree tertile (Table 2). Overall, use of AADs decreased over time and rural–urban differences between them were observed across the study period, although the differences were nonsignificant in the last three years of follow-up (Figure 2). Regarding specific AADs, patients with urban residence were more likely to receive flecainide, dronedarone, amiodarone, and sotalol than patients with rural residence (Supplementary Table S2).

### 3.3. Cardioversions

Overall, 45 868 cardioversion procedures were performed in 28,132 (15.8%) patients. While no disparities in the adjusted rate of cardioversion procedures were observed according to the rural–urban status, the highest urbanization degree tertile was associated with a higher rate of these procedures (Table 2). Patients with urban residence were more likely to undergo more than one cardioversion (Supplementary Table S2). The annual rural–urban disparities in the performance of cardioversion within one-year follow-up were inconsistent and not statistically significant (Figure 2).

### 3.4. Catheter Ablations

Altogether, 4711 catheter ablation procedures were performed on 3872 (2.2%) patients during 2010–2018. Although the adjusted catheter ablation incidence rate did not differ between patients with rural and urban residence, the highest urbanization degree tertile was associated with higher ablation rate. (Table 2). Likelihood of repeat ablation procedures was higher in patients with more urban residence (Supplementary Table S2). While the overall use of catheter ablation increased steadily during 2010–2018, the annual rural–urban differences did not reach statistical significance (Figure 2).

## 4. Discussion

This retrospective cohort study covering all patients with incident AF in Finland documented marginal rural–urban disparities in the use of rhythm control therapies. Although the differences in AAT use were largely nonsignificant when patients were stratified according to the rural–urban classification system, slightly higher use of any AAT, AADs, cardioversion, and catheter ablation procedures were observed in the highest urbanization degree tertile when compared to the lowest tertile.

Previous research addressing rural–urban disparities in the use of rhythm control therapies in patients with AF is limited and has provided somewhat inconsistent results. A recent study conducted in Norway among patients with AF diagnosed in specialist health care observed a lower use of catheter ablation in the norther, more rural, regions [24]. Additionally, a study conducted in Canada among patients with new-onset AF during 2010–2012 reported that patients with rural residence have a lower likelihood of electrophysiologist assessment, but paradoxically a higher rate of AF ablation procedures than patients with urban residence [25]. However, these prior works may have been prone to significant selection and confounding biases owing to inclusion of only patients diagnosed with AF in the specialist care and limited controlling for comorbidities and other patient characteristics. Moreover, no study has covered all modalities of rhythm control therapy. Therefore, the results of the current study, based on comprehensive data on all patients with AF in Finland from all levels of care, provide substantially more solid evidence and increase our understanding on this topic. AATs were not used in a vast majority of patients (79%), indicating that rate control predominated as the chosen treatment approach over rhythm control strategy. The overall need of AATs in our nationwide study cohort is likely reduced by the relatively high mean age and coverage of all types of AF, including patients with self-limiting, infrequent, or asymptomatic AF episodes, as well as, uniquely, patients treated solely in primary care.

The largest disparities were observed in the use of AADs, 18% higher adjusted incidence rate in the highest urbanization degree tertile, when compared to the lowest. Indeed, AAD use was the only AAT category, in which a disparity was observed also between patients stratified according to the rural–urban classification. Overall, especially apart from AAD use, the observed differences in the use of AATs were only marginal, suggesting that no large inequity exists between rural and urban areas in the patient selection and utilization of AATs in Finland. These findings are somewhat discordant with several previous reports of lower quality of care for cardiovascular diseases in rural areas [10,26,27].

Nevertheless, small differences in the utilization of AATs were observed, with the underlying causes likely being multifactorial. Patients from more rural areas may have a higher threshold in seeking care for arrhythmia-related symptoms, and poorer access to healthcare services may further hamper patients from receiving symptomatic treatment in rural areas. Furthermore, the higher availability of private sector and specialist care in urban areas may increase prescriptions of AADs, as well as indirectly the use of cardioversion and ablation procedures through higher rate of hospital referrals. Additionally, both patient advocacy and preference for more intensive or invasive AATs may differ between patients residing in rural and urban areas. Finally, the higher prevalence of cardiovascular comorbidities and lower income in rural areas likely impact the clinical decision making of AATs, although these factors were controlled for in our adjusted analyses. Indeed, low income has been previously associated with lower use of rhythm control therapies [28]. 

Our results must be interpreted bearing in mind the limitations of this study, chiefly the challenges inherent to the retrospective cohort study design and use of administrative data. Thus, our findings represent associations and not necessarily causality. Moreover, information bias may be present due to inaccurately recorded data in the used health care registers. Although our analyses were adjusted for several patient characteristics, residual confounding cannot be excluded. Varying rural–urban definitions exist in previous literature, and additionally, peculiar differences in residence distribution between countries may hinder the generalizability of our findings. Furthermore, we lacked data on AF symptoms, AF subclassifications, whether the patient had atrial flutter or fibrillation, and the actual reasons for withholding AATs. Therefore, we are unable to definitively distinguish, whether our findings represent underuse or overuse of AATs in certain areas. Additionally, expect for diagnosed alcohol use disorders, we lacked information on other lifestyle-related factors. Despite these limitations, the particular strengths of the current study are the large nationwide sample size covering all levels of care, the primary care, and comprehensive data on comorbidities and individual-level socioeconomic factors, enabling extensive adjusting for confounders.

## 5. Conclusions

In conclusion, we observed slightly higher use of AATs among patients with AF residing in urban areas than among those with rural residence. The rural–urban differences were most evident in the use of AADs. Otherwise, the magnitude of the observed disparities was relatively low, suggesting that in the overall utilization of rhythm control strategy, no large rural–urban inequity exists in Finland.

## Figures and Tables

**Table 1:** Descriptive characteristics of the cohort.

	Rural–Urban Status			Urbanization Degree Tertiles			
	Rural	Urban	*p*-Value	1st (lowest)	2nd	3rd (highest)	*p*-Value
	*n* = 62,836	*n* = 114,693		*n* = 59,069	*n* = 59,531	*n* = 58,929	
**Demographics**							
Mean age, years	73.7 (12.5)	72.7 (13.2)	<0.001	73.9 (12.4)	72.7 (13.1)	72.6 (13.4)	<0.001
Female sex	30,715 (48.9)	57,880 (50.5)	<0.001	29,045 (49.2)	29,731 (49.9)	29,819 (50.6)	<0.001
**Highest educational level**			<0.001				<0.001
Primary school	35,869 (57.1)	53,964 (47.1)		34,205 (57.9)	29,612 (49.7)	26,016 (44.1)	
Upper secondary school	18,076 (28.8)	31,678 (27.6)		16,883 (28.6)	17,288 (29.0)	15,583 (26.4)	
Higher education	8891 (14.1)	29,051 (25.3)		7981 (13.5)	12,631 (21.2)	17,330 (29.4)	
**Income quartiles**			<0.001				<0.001
1st (lowest)	19,905 (31.7)	24,250 (21.1)		19,234 (32.6)	14,027 (23.6)	10,894 (18.5)	
2nd	16,698 (26.6)	28,166 (24.6)		15,815 (26.8)	15,618 (26.2)	13,431 (22.8)	
3rd	14,316 (22.8)	29,838 (26.0)		13,207 (22.4)	15,542 (26.1)	15,405 (26.1)	
4th (highest)	11,917 (19.0)	32,439 (28.3)		10,813 (18.3)	14,344 (24.1)	19,199 (32.6)	
**Comorbidities**							
Abnormal liver function	299 (0.5)	656 (0.6)	0.008	267 (0.5)	312 (0.5)	376 (0.6)	<0.001
Abnormal renal function	2650 (4.2)	5221 (4.6)	<0.001	2500 (4.2)	2556 (4.3)	2815 (4.8)	<0.001
Alcohol use disorder	2610 (4.2)	5263 (4.6)	<0.001	2497 (4.2)	2561 (4.3)	2815 (4.8)	<0.001
Cancer	12,731 (20.3)	24,966 (21.8)	<0.001	11,929 (20.2)	12,295 (20.7)	13,473 (22.9)	<0.001
Coronary artery disease	15,256 (24.3)	25,542 (22.3)	<0.001	14,421 (24.4)	14,006 (23.5)	12,371 (21.9)	<0.001
Dementia	3383 (5.4)	6258 (5.5)	0.519	3247 (5.5)	3126 (5.3)	3268 (5.5)	0.056
Diabetes	14,926 (23.8)	25,853 (22.5)	<0.001	14,113 (23.9)	13,675 (23.0)	12,991 (22.0)	<0.001
Dyslipidemia	32,120 (51.1)	58,131 (50.7)	0.081	30,322 (51.3)	30,690 (51.6)	29,239 (49.6)	<0.001
Heart failure	11,365 (18.1)	19,350 (16.9)	<0.001	10,914 (18.5)	10,209 (17.1)	9592 (16.3)	<0.001
Hypertension	48,263 (76.8)	86,780 (75.7)	<0.001	45,423 (76.9)	45,197 (75.9)	44,423 (75.4)	<0.001
Prior bleeding	7166 (11.4)	13,339 (11.6)	0.154	6775 (11.5)	6898 (11.6)	6832 (11.6)	0.754
Prior ischemic stroke	7403 (11.8)	13,052 (11.4)	0.011	7063 (12.0)	6789 (11.4)	6603 (11.2)	<0.001
Prior myocardial infarction	6096 (9.7)	9893 (8.6)	<0.001	5796 (9.8)	5398 (9.1)	4795 (8.1)	<0.001
Psychiatric disorder	8891 (14.1)	17,977 (15.7)	<0.001	8462 (14.3)	9060 (15.2)	9346 (15.9)	<0.001
**Risk scores**							
Modified HAS-BLED score	2.6 (1.0)	2.6 (1.0)	0.007	2.6 (1.0)	2.6 (1.0)	2.6 (1.1)	<0.001
CHA_2_DS_2_-VASc score	3.6 (1.9)	3.5 (1.9)	<0.001	3.6 (1.9)	3.5 (1.9)	3.4 (1.9)	<0.001

Values denote *n* (%) or mean (standard deviation). Abbreviations: CHA_2_DS_2_-VASc, congestive heart failure, hypertension, age ≥ 75 years, diabetes, history of stroke or transient ischemic attack, vascular disease, age 65–74 years, sex category (female); modified HAS-BLED score, hypertension, abnormal renal or liver function, prior stroke, bleeding history, age > 65 years, alcohol abuse, concomitant antiplatelet/NSAIDs (no labile INR, max score 8).

**Table 2:** Incidence of AAT use during follow-up according to rural–urban status and urbanization degree tertiles.

**Outcome**	**Residence**	**Interventions** ***n* (%)**	**Patient Years (in 1000 years)**	**Incidence Rate (per 1000 patient years**	**Unadjusted IRR**	**Adjusted IRR**
Any AAT	Rural	12,361 (19.7)	163	75.8 (74.4–77.1)	(Reference)	(Reference)
	Urban	24,307 (21.2)	299	81.3 (80.3–82.4)	1.07 (1.05–1.10)	1.01 (0.99–1.03)
AADs	Rural	4407 (7.0)	192	22.9 (22.2–23.6)	(Reference)	(Reference)
	Urban	9636 (8.4)	352	27.4 (26.8–27.9)	1.20 (1.16–1.24)	1.11 (1.07–1.15)
Cardioversion	Rural	9543 (15.2)	174	54.9 (53.8–56.0)	(Reference)	(Reference)
	Urban	18,589 (16.2)	321	58.0 (57.2–58.8)	1.06 (1.03–1.08)	1.01 (0.98–1.03)
Catheter ablation	Rural	1195 (1.9)	206	5.8 (5.5–6.1)	(Reference)	(Reference)
	Urban	2677 (2.3)	381	7.0 (6.8–7.3)	1.21 (1.13–1.30)	1.05 (0.98–1.12)
**Outcome**	**Urbanization Degree Tertiles**	**Interventions** ***n* (%)**	**Patient Years (in 1000 years)**	**Incidence Rate (per 1000 patient years**	**Unadjusted IRR**	**Adjusted IRR**
Any AAT	1st	11,308 (19.1)	154	73.7 (72.3–75.0)	(Reference)	(Reference)
	2nd	12,506 (21.0)	156	79.9 (78.5–81.3)	1.09 (1.06–1.11)	1.01 (0.99–1.04)
	3rd	12,854 (21.8)	152	84.6 (83.2–86.1)	1.15 (1.12–1.18)	1.06 (1.03–1.08)
AADs	1st	4002 (6.8)	180	22.2 (21.5–22.9)	(Reference)	(Reference)
	2nd	4795 (8.1)	185	25.9 (25.2–26.7)	1.17 (1.12–1.22)	1.07 (1.03–1.12)
	3rd	5246 (8.9)	179	29.3 (28.5–30.1)	1.32 (1.27–1.37)	1.18 (1.14–1.23)
Cardioversion	1st	8748 (14.8)	163	53.6 (52.5–54.7)	(Reference)	(Reference)
	2nd	9567 (16.1)	168	57.1 (55.9–58.2)	1.07 (1.03–1.10)	1.01 (0.98–1.04)
	3rd	9817 (16.7)	164	60.0 (58.8–61.2)	1.12 (1.09–1.15)	1.05 (1.02–1.08)
Catheter ablation	1st	1052 (1.8)	192	5.5 (5.2–5.8)	(Reference)	(Reference)
	2nd	1378 (2.3)	199	6.9 (6.6–7.3)	1.27 (1.17–1.37)	1.09 (1.00–1.18)
	3rd	1442 (2.4)	196	7.4 (7.0–7.8)	1.35 (1.24–1.46)	1.10 (1.02–1.19)

Abbreviations: AAD, antiarrhythmic drug; AAT, antiarrhythmic therapy; IRR, incidence rate ratio. 95% confidence intervals in parenthesis. IRRs estimated by Poisson regression and adjusted for age, sex, calendar year of AF diagnosis, education level, income quartiles, dementia, cancer, alcohol use disorder, psychiatric disorders, prior stroke, abnormal liver function, abnormal kidney function, diabetes, hypertension, coronary heart disease, and heart failure.

## Data Availability

Because of the sensitive nature of the data collected for this study, requests to access the dataset from qualified researchers trained in human subject confidentiality protocols may be sent to the Finnish national register holders (KELA, Finnish Institute for Health and Welfare, Population Register Center and Tax Register) through Findata (https://findata.fi/en/).

## Supplementary Materials

The following supporting information can be downloaded at: https://www.mdpi.com/article/10.3390/ijerph191811191/s1, Table S1: Definitions of the comorbidities; Table S2: Use of AADs and repeat AAT procedures during follow-up; Figure S1: Flow-chart of the patient selection process.

## References

[1] Björk S., Palaszewski B., Friberg L., Bergfeldt L. (2013). Atrial fibrillation, stroke risk, and warfarin therapy revisited: A population-based study. Stroke.

[2] Lehto M., Halminen O., Mustonen P., Putaala J., Linna M., Kinnunen J., Kouki E., Niiranen J., Hartikainen J., Haukka J. (2022). The nationwide Finnish anticoagulation in atrial fibrillation (FinACAF): Study rationale, design, and patient characteristics. Eur. J. Epidemiol..

[3] Potpara T., Dagres N., Arbelo E., Bax J.J., Blomstrom-Lundqvist C. (2021). 2020 ESC Guidelines for the diagnosis and management of atrial fibrillation developed in collaboration with the European Association for Cardio-Thoracic Surgery (EACTS). Eur. Heart J..

[4] Kirchhof P., Camm A.J., Goette A., Brandes A., Eckardt L., Elvan A., Fetsch T., van Gelder I.C., Haase D., Haegeli L.M. (2020). Early Rhythm-Control Therapy in Patients with Atrial Fibrillation. N. Engl. J. Med..

[5] Al Halabi S., Qintar M., Hussein A., Alraies M.C., Jones D.G., Wong T., MacDonald M.R., Petrie M.C., Cantillon D., Tarakji K.G. (2015). Catheter ablation for atrial fibrillation in heart failure patients: A meta-analysis of randomized, controlled trials. JACC Clin. Electrophysiol..

[6] Marrouche N.F., Brachmann J., Andresen D., Siebels J., Boersma L., Jordaens L., Merkely B., Pokushalov E., Sanders P., Proff J. (2018). Catheter Ablation for Atrial Fibrillation with Heart Failure. N. Engl. J. Med..

[7] Kapral M.K., Austin P.C., Jeyakumar G., Hall R., Chu A., Khan A.M., Jin A.Y., Martin C., Manuel D., Silver F.L. (2019). Rural-urban differences in stroke risk factors, incidence, and mortality in people with and without prior stroke: The CANHEART stroke study. Circ. Cardiovasc. Qual. Outcomes.

[8] Teng T.H.K., Katzenellenbogen J.M., Hung J., Knuiman M., Sanfilippo F.M., Geelhoed E., Hobbs M., Thompson S.C. (2014). Rural-urban differentials in 30-day and 1-year mortality following first-ever heart failure hospitalisation in Western Australia: A population-based study using data linkage. BMJ Open.

[9] Braveman P., Gottlieb L. (2014). Urban-rural differences in coronary heart disease mortality in the United States: 1999–2009. Public Health Rep..

[10] Supina A.L., Guirguis L.M., Majumdar S.R., Lewanczuk R.Z., Lee T.K., Toth E.L., Johnson J.A. (2004). Treatment gaps for hypertension management in rural canadian patients with type 2 diabetes mellitus. Clin. Ther..

[11] Tran C., Wijeysundera H.C., Qui F., Tu J.V., Bhatia R.S. (2014). Comparing the ambulatory care and outcomes for rural and urban patients with chronic ischemic heart disease: A population-based cohort study. Circ. Cardiovasc. Qual. Outcomes.

[12] Bray B.D., Paley L., Hoffman A., James M., Gompertz P., Wolfe C.D., Hemingway H., Rudd A.G., SSNAP Collaboration (2018). Socioeconomic disparities in first stroke incidence, quality of care, and survival: A nationwide registry-based cohort study of 44 million adults in England. Lancet Public Health.

[13] Gamble J.M., Eurich D.T., Ezekowitz J.A., Kaul P., Quan H., McAlister F.A. (2011). Patterns of care and outcomes differ for urban versus rural patients with newly diagnosed heart failure, even in a universal healthcare system. Circ.: Heart Fail..

[14] Flaker G.C., McGowan D.J., Boechler M., Fortune G., Gage B. (1999). Underutilization of antithrombotic therapy in elderly rural patients with atrial fibrillation. Am. Heart J..

[15] Dalmau Llorca M.R., Aguilar Martín C., Carrasco-Querol N., Hernández Rojas Z., Forcadell Drago E., Rodríguez Cumplido D., Castro Blanco E., Pepió Vilaubí J.M., Gonçalves A.Q., Fernández-Sáez J. (2021). Gender and socioeconomic inequality in the prescription of direct oral anticoagulants in patients with non-valvular atrial fibrillation in primary care in catalonia (Fantas-TIC study). Int. J. Environ. Res. Public Health.

[16] Wu C., McMurtry M.S., Sandhu R.K., Youngson E., Ezekowitz J.A., Kaul P., McAlister F.A. (2015). Impact of rural residence on warfarin use and clinical events in patients with non-valvular atrial fibrillation: A Canadian population based study. PLoS ONE.

[17] Avgil Tsadok M., Jackevicius C.A., Essebag V., Eisenberg M.J., Rahme E., Pilote L. (2015). Warfarin Treatment and Outcomes of Patients With Atrial Fibrillation in Rural and Urban Settings. J. Rural Health.

[18] O’Neal W.T., Sandesara P.B., Kelli H.M., Venkatesh S., Soliman E.Z. (2018). Urban-rural differences in mortality for atrial fibrillation hospitalizations in the United States. Heart Rhythm.

[19] Jaakkola J., Teppo K., Biancari F., Halminen O., Putaala J., Mustonen P., Haukka J., Linna M., Kinnunen J., Tiili P. (2021). The effect of mental health conditions on the use of oral anticoagulation therapy in patients with atrial fibrillation: The FinACAF Study. Eur. Heart J. Qual. Care Clin. Outcomes.

[20] Teppo K., Jaakkola J., Airaksinen K.J., Biancari F., Halminen O., Putaala J., Mustonen P., Haukka J., Hartikainen J., Luojus A. (2022). Mental health conditions and adherence to direct oral anticoagulants in patients with incident atrial fibrillation: A nationwide cohort study. Gen. Hosp. Psychiatry.

[21] Teppo K., Jaakkola J., Biancari F., Halminen O., Putaala J., Mustonen P., Haukka J., Linna M., Kinnunen J., Tiili P. (2022). Mental health conditions and risk of first-ever ischaemic stroke and death in patients with incident atrial fibrillation: A nationwide cohort study. Eur. J. Clin. Investig..

[22] Finland’s Environmental Administration Urban-Rural Classification. https://www.ymparisto.fi/fi-FI/Elinymparisto_ja_kaavoitus/Yhdyskuntarakenne/Tietoa_yhdyskuntarakenteesta/Kaupunkimaaseutu_luokitus.

[23] Statistics Finland Degree of Urbanization. https://pxnet2.stat.fi/PXWeb/pxweb/en/StatFin/StatFin__vrm/.

[24] Olsen F., Uleberg B., Jacobsen B.K., Heuch I., Tande P.M., Bugge E., Balteskard L. (2022). Socioeconomic and geographic differences in ablation of atrial fibrillation in Norway—A national cohort study. BMC Public Health.

[25] Singh S.M., Webster L., Ko D.T., Tu J.V., Wijeysundera H.C. (2017). Factors Associated With Cardiac Electrophysiologist Assessment and Catheter Ablation Procedures in Patients with Atrial Fibrillation. JACC Clin. Electrophysiol..

[26] Loccoh E.C., Joynt Maddox K.E., Wang Y., Kazi D.S., Yeh R.W., Wadhera R.K. (2022). Rural-Urban Disparities in Outcomes of Myocardial Infarction, Heart Failure, and Stroke in the United States. J. Am. Coll. Cardiol..

[27] Hammond G., Luke A.A., Elson L., Towfighi A., Joynt Maddox K.E. (2020). Urban-Rural Inequities in Acute Stroke Care and In-Hospital Mortality. Stroke.

[28] Teppo K., Jaakkola J., Biancari F., Halminen O., Linna M., Haukka J., Putaala J., Mustonen P., Kinnunen J., Luojus A. (2022). Socioeconomic disparities in use of rhythm control therapies in patients with incident atrial fibrillation: A Finnish nationwide cohort study. IJC Heart Vasc..

