# Supplementary material for: Rural–Urban differences in Use of Rhythm Control Therapies in Patients with Incident Atrial Fibrillation: A Finnish Nationwide Cohort Study
Source: Int J Environ Res Public Health. 2022 Sep 6;19(18):11191. doi: 10.3390/ijerph191811191 (PMC9517432; doi:10.3390/ijerph191811191)

Supplementary Material

Supplementary Table S1. Definitions of the comorbidities

Supplementary Table S2. Use of AADs and repeat AAT procedures during follow-up

Supplementary Figure S1. Flow-chart of the patient selection process

Supplementary Table S1. Definitions of the comorbidities

	ICD-10	ICPC-2	Reimbursement code	ATC code	Other
Hypertension	I10-I15	K85, K86, K87	205	C03A, C03B, C03DB, C03EA, C07A, C08CA, C08D, C09	
Dyslipidemia	E78	T93	206	C10	
Heart failure	I50, I11.0, I13.0, I13.2	K77	201		
Diabetes	E10-E14	T89, T90	103, 215	A10	
Previous stroke	I63, I64, I69.3-I69.8	K90			
Bleeding history	D50.0, D62, D68.3, I60-I62, I69.0-I69.2, I85.0, I86.4, J94.2, K22.1, K22.3, K22.6, K25.0, K25.2, K25.4, K25.6, K26.0, K26.2, K26.4, K26.6, K27.0, K27.2, K27.4, K27.6, K28.0, K28.2, K28.4, K28.6, K29.0, K62.5, K63.1, K63.3, K92.0-K92.2, N02, R04, R31, R58, S06.2-S06.6, S06.8				
Alcohol abuse	F10				
Renal failure or dialysis	N18, Z49				
Liver cirrhosis or failure	K70.2-K70.4, K71.7, K71.8, K72, K74				
Dementia	F00-F03, G30				
Cancer					Any cancer registered in the Finnish Cancer Registry
Coronary heart disease	I21-I25				
Prior myocardial infarction	I21-I22				
Psychiatric disorder	F04-F99				

Abbreviations: ATC, anatomic therapeutic chemical; ICD-10, International Classification of Diseases, Tenth Revision; ICPC-2, International Classification of Primary Care, Second Edition

Supplementary table S2. Use of AADs and repeat AAT procedures during follow-up

	Rural-urban status			Urbanization degree tertiles			
	**Rural**	**Urban**	**P-value**	**1st (lowest)**	**2nd**	**3rd (highest)**	**P-value**
Amiodarone	1 489 (2.4)	3 074 (2.7)	<0.001	1 310 (2.2)	1 545 (2.6)	1 708 (2.9)	<0.001
Dronedarone	214 (0.3)	489 (0.4)	0.006	187 (0.3)	256 (0.4)	260 (0.4)	<0.001
Flecainide	2 317 (3.7)	5 082 (4.4)	<0.001	2 179 (3.7)	2 593 (4.4)	2 627 (4.5)	<0.001
Sotalol	356 (0.6)	928 (0.8)	<0.001	295 (0.5)	372 (0.6)	617 (1.0)	<0.001
Ablations >1	223 (0.4)	477 (0.4)	0.050	119 (0.3)	245 (0.4)	256 (0.4)	0.020
Cardioversions > 1	2 680 (4.3)	5 549 (4.8)	<0.001	2 420 (4.1)	2 864 (4.8)	2 945 (5.0)	<0.001

Supplementary Figure S1. Flow-chart of the patient selection process

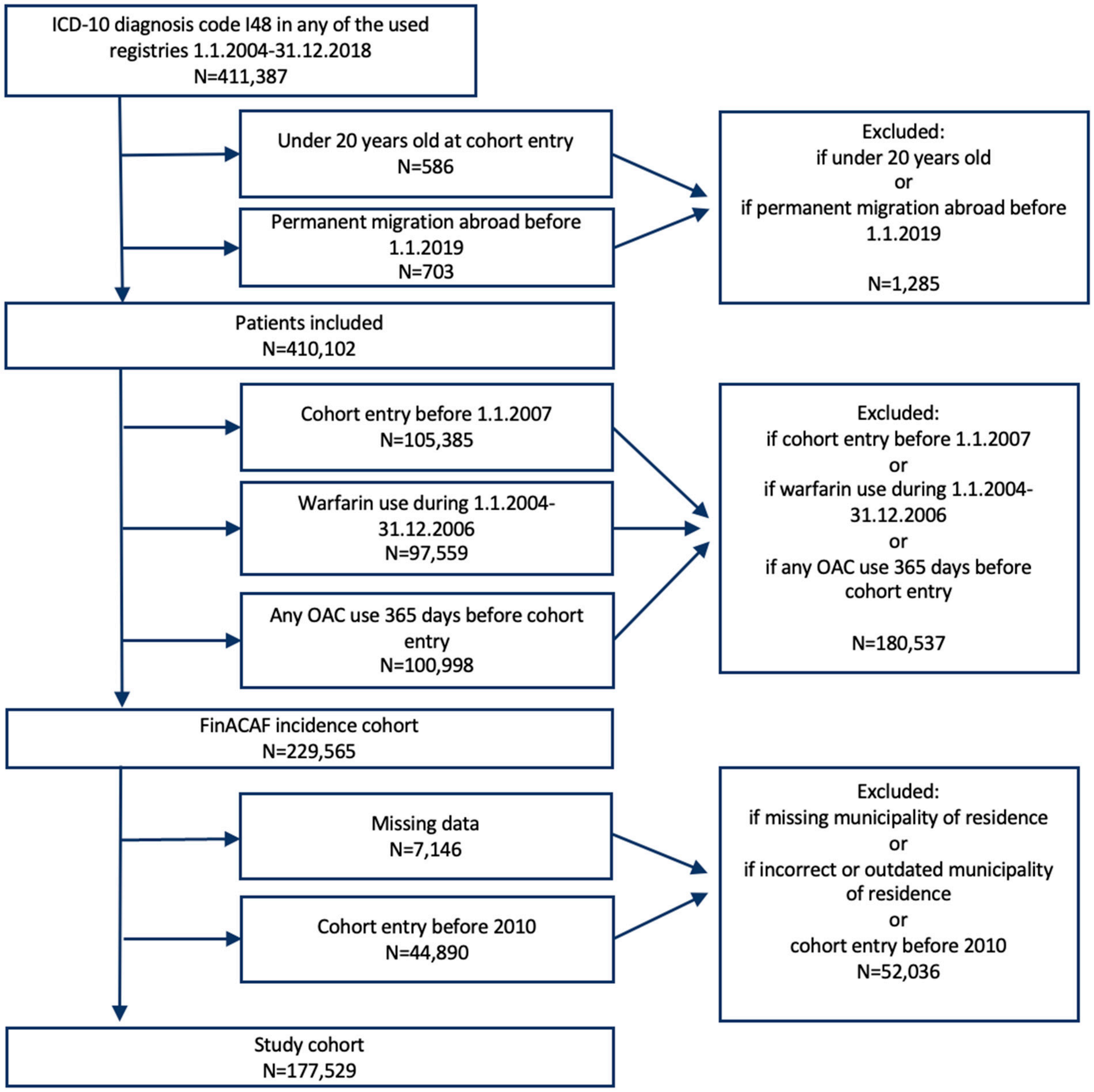

Supplement: Supplementary file 1 [file ijerph-19-11191-s001.zip › ijerph-1874184-supplementary.pdf]
